# Isolation and characterization of an antimicrobial *Bacillus subtilis* strain O-741 against *Vibrio parahaemolyticus*

**DOI:** 10.1371/journal.pone.0299015

**Published:** 2024-04-04

**Authors:** Yi-An Chen, Wen-Chin Chiu, Tzu-Yun Wang, Hin-chung Wong, Chung-Tao Tang

**Affiliations:** 1 Department of Microbiology, Soochow University, Taipei, Taiwan, Republic of China; 2 School of Medicine, College of Medicine, I-Shou University, Kaohsiung, Taiwan, Republic of China; 3 School of Medicine for International Students, College of Medicine, I-Shou University, Kaohsiung, Taiwan, Republic of China; Tanta University Faculty of Agriculture, EGYPT

## Abstract

*Vibrio parahaemolyticus* is a marine bacterium that can infect and cause the death of aquatic organisms. *V*. *parahaemolyticus* can also cause human foodborne infection via contaminated seafood, with clinical syndromes which include diarrhea, abdominal cramps, nausea and so on. Since controlling *V*. *parahaemolyticus* is important for aquaculture and human health, various strategies have been explored. This study investigates the application of antagonistic microorganisms to inhibit the growth of *V*. *parahaemolyticus*. We screened aquaculture environment samples and identified a *Bacillus subtilis* strain O-741 with potent antimicrobial activities. This strain showed a broad spectrum of antagonistic activities against *V*. *parahaemolyticus* and other *Vibrio* species. Application of the O-741 bacterium significantly increased the survival of *Artemia* nauplii which were infected with *V*. *parahaemolyticus*. Furthermore, the cell-free supernatant (CFS) of O-741 bacterium exhibited inhibitory ability against *V*. *parahaemolyticus*, and its activity was stable to heat, acidity, UV, enzymes, and organic solvents. Next, the O-741 CFS was extracted by ethyl acetate, and analyzed by ultra-performance liquid chromatography-mass-mass spectrometry (UPLC-MS/MS), and the functional faction was identified as an amicoumacin A compound. The organic extracts of CFS containing amicoumacin A had bactericidal effects on *V*. *parahaemolyticus*, and the treated *V*. *parahaemolyticus* cells showed disruption of the cell membrane and formation of cell cavities. These findings indicate that *B*. *subtilis* strain O-741 can inhibit the *V*. *parahaemolyticus in vitro* and *in vivo*, and has potential for use as a biocontrol agent for preventing *V*. *parahaemolyticus* infection.

## Introduction

*Vibrio parahaemolyticus* is a prevalent foodborne pathogen in Taiwan and is a cause of gastroenteritis in many Asian countries [1]. It is a gram-negative and halophilic bacterium that is widely disseminated in estuarine, marine and coastal environments [2]. By consumption of contaminated raw or undercooked seafood, *V*. *parahaemolyticus* can cause human infection. *V*. *parahaemolyticus* is currently classified into 13 O serotypes and 71 K serotypes [3]. Since the occurrence of pandemic O3:K6 strains in 1996, *V*. *parahaemolyticus* has gained global significance [4]. Typically, the clinical isolates of *V*. *parahaemolyticus* express thermostable direct hemolysin (TDH) and produce ß-hemolysis on Wagatsuma agar, which is known as the Kanagawa phenomenon (KP) positive [5]. Some KP-negative *V*. *parahaemolyticus* isolates are hemolytic and contain TDH-related hemolysin (TRH). TDH and TRH are the main virulence factors of *V*. *parahaemolyticus* [6].

In addition, *V*. *parahaemolyticus* can survive in fish and shellfish aquaculture, and cause infections in some cultured shrimps [7]. Acute hepatopancreatic necrosis disease (AHPND), caused by this bacterium, is a severe shrimp disease that can lead to mortality and substantial economic loss [8, 9]. Thus, the prevention of *V*. *parahaemolyticus* infection is beneficial to aquaculture. Among several approaches investigated so far, applications of antagonistic microorganisms, disinfectants, antibiotics, antimicrobial peptides, botanical extracts, or lytic bacteriophages have been evaluated for control of this pathogen [10–15]. As antagonistic bacteria, *Bacillus* species are widely distributed in nature, including marine environments, and are safe for use as probiotics [16–18]. Some *Bacillus* species can express active natural products and exhibit a wide spectrum of antimicrobial activities against pathogenic bacteria [19, 20]. Therefore, *Bacillus* species are regarded as appropriate biological control agent (BCA) candidates for treating bacterial infections [17].

Recently, the significance of pathogenic *V*. *parahaemolyticus* in aquaculture and human health has increased [21, 22], and aquaculture samples are a prominent source of this pathogen [23]. It is increasingly necessary to control the risk of *V*. *parahaemolyticus* in aquaculture and the use of antagonistic bacteria seems like a promising option. In this study, we screened and identified an antimicrobial *Bacillus subtilis* strain O-741, and characterized its activity, stability and targeted *V*. *parahaemolyticus* cell response. Furthermore, its active antimicrobial compound was identified and the application of this strain was evaluated *in vivo* using *Artemia* nauplii. The results indicated that O-741 bacterium may be useful as a biocontrol agent against *V*. *parahaemolyticus*.

## Materials and methods

### Strains and culture conditions

The *Vibrio* spp. used in this study are listed in Table 1. *V*. *chloreae* strains were stored in Luria Bertani (LB) broth with 30% glycerol (v/v). Other *Vibrio* spp. were stored in the same broth with 3% NaCl (LB-3% NaCl), and these bacterial strains were stocked at -80°C. The strains were recovered from frozen stocks and cultured in LB broth or LB-3% NaCl at 37°C with shaking at 160 rpm for 16–18 hours.

**Table 1:** Bacterial strains used in this study.

*Vibrio* species	Strain no.	Description	Source	Reference
*V*. *parahaemolyticus*	2008–1198	Serotype O4:K8, clinical isolate	CDC, Taiwan	[48]
*V*. *parahaemolyticus*	DON 1259	Serotype O1:K25, clinical isolate	Thailand	-
*V*. *parahaemolyticus*	DON 1362	Serotype O4:K68, clinical isolate	CDC, Taiwan	-
*V*. *parahaemolyticus*	KX-V231	Serotype O3:K6, clinical isolate	Thailand	[48]
*V*. *parahaemolyticus*	RIMD2210633	Serotype O3:K6, clinical isolate	Japan	[55]
*V*. *parahaemolyticus*	ATCC 27969	Serotype unknown, environmental isolate	ATCC	[56]
*V*. *parahaemolyticus*	BC100620-2	Serotype O10:KUT, environmental isolate	Clam, Tainan Beimen	[48]
*V*. *parahaemolyticus*	CS090909-4	None detected, environmental isolate	Soil sample, Tainan Qigu	[48]
*V*. *parahaemolyticus*	D/4	Serotype unknown, environmental isolate	Fish pathogen, National Cheng Kung University	[8]
*V*. *parahaemolyticus*	DO091211-3	Serotype O5:K43, environmental isolate	Oyster, Chiayi Dongshi	[48]
*V*. *parahaemolyticus*	FC090912-2	None detected, environmental isolate	Clam, Changhua Fangyuan	[48]
*V*. *parahaemolyticus*	SCS1112-1	Serotype unknown, environmental isolate	Bottom mud of fish pond, Tainan Shuangchun	[57]
*V*. *parahaemolyticus*	SCS1112-2	Serotype unknown, environmental isolate	Bottom mud of fish pond, Tainan Shuangchun	[57]
*V*. *parahaemolyticus*	SW090307-6	Serotype O8:K43, environmental isolate	Water sample, Changhua Shengang	[48]
*V*. *parahaemolyticus*	YAS1206-16	None detected, environmental isolate	Soil sample, Tainan Yongan	[15]
*V*. *anguillarum*	ATCC 19265	environmental isolate	ATCC	[58]
*V*. *alginolyticus*	ATCC 17749	environmental isolate	Spoiled horse mackerel, Japan	[59]
*V*. *chloreae*	NIH 35A3	Serotype O10:KUT, environmental isolate	Inaba, NIPN Y.S.L	[60]
*V*. *chloreae*	NIH 41	environmental isolate	Ogawa, NIPN Y.S.L	[60]
*V*. *fluvialis*	ATCC 33809	Serotype O5:K43, environmental isolate	Human feces, Dacca, Bangladesh	[61]
*V*. *harveyi*	ATCC 14126	environmental isolate	Dead luminescing amphipod, Massachusetts	[62]
*V*. *harveyi*	S14	environmental isolate	Tainan pond water	-
*V*. *vulnificus*	B5	environmental isolate	Tainan pond water	-

Abbreviations: RIMD, Research Institute for Microbial Diseases; ATCC, American Type Culture Collection; NIH, National Institutes of Health; CDC, Centers for Disease Control.

### Isolation and screening of antimicrobial bacteria

A total of 1,545 bacterial isolates were isolated from fish gills, fish intestines, oysters and clams, which were collected within the cold chain of a fish market. The samples were homogenized, suspended in phosphate buffered saline (PBS), streaked on Tryptic Soy Agar (TSA)-3% NaCl plates, and incubated at room temperature for 1–2 days. The isolated colonies were screened for antimicrobial activities by spot inoculation on bacterial lawn with indicator bacteria. The *V*. *parahaemolyticus* strains D/4, KX-V231, *V*. *harveyi* strain S14, and *V*. *vulnificus* strain B5 were used as indicators.

### Identification of antimicrobial O-741 bacterium

The O-741 bacterium which was isolated from oyster was cultured in LB for 16–18 hours, and bacterial cells were harvested by centrifugation. The genomic DNA from the cell pellet was extracted using a commercial DNA extraction kit (Genomic DNA Mini Kit, Geneaid Biotech). The 16S rRNA, *gyrA* and *rpoB* genes of the genomic DNA were amplified by polymerase chain reactions (PCR) with the primers shown in Table 2 [24, 25]. After DNA sequencing, the nucleotide sequences of amplified fragments were applied to homology search using the BLAST software of the NCBI. The phylogenetic trees were built in MEGA6, using the neighbor-joining method [11, 26, 27]. The bootstrap values were calculated based on 1000 computer-generated trees.

**Table 2:** Primers used to identify the O-741 bacterium.

Designation	Sequence (5’ ->3’)	Target	Amplicon, bp	Reference
16S 8F	AGAGTTTGATCCTGGCTCAG	16S rRNA gene	1,493	[24]
16S 1500R	AGAAAGGAGGTGATCCAGCC			
*gyrA*-F	CAGTCAGGAAATGCGTACGTCCTT	*gyrA* gene	1,028	[25]
*gyrA*-R	CAAGGTAATGCTCCAGGCATTGCT			
*rpoB*-F	AGGTCAACTAGTTCAGTATGGAC	*rpoB* gene	580	[25]
*rpoB*-R	AAGAACCGTAACCGGCAACTT			

Abbreviations: F, forward; R, reverse; gyrA, gyrase subunit A; rpoB, RNA polymerase beta subunit.

### Evaluation of antimicrobial activities of O-741 bacterium

The O-741 bacterium was grown in 100 ml LB broth at 37°C, 160 rpm for 24, 48, and 72 hours and the bacterial cultures were separated into three fractions, the untreated bacterial cultures, bacterial pellets resuspended in LB broth, and cell-free supernatants (CFS). The CFS was collected by centrifugation at 16,000 rpm for 1 min and filtered through 0.22 μm polyethersulfone membrane (Merck Millipore, Ireland). The antagonistic activities of the prepared fractions were tested by well diffusion assays [28]. Aliquots of 30 μl of the prepared fractions were added into 6-mm wells on LB-3% NaCl agar with different bacterial lawns (Table 1), incubated at 37°C for 24 hours, and the diameters of the inhibition zones were measured.

### *In vivo* challenge with the *Artemia* nauplii model

Axenic *Artemia* nauplii were obtained by a decapsulation and hatching process. Two hundred milligrams of *Artemia* cysts (Ocean Star International, Snowville, UT) were hydrated in double-distilled water (ddH_2_O) for 1 hour. Then, the sterile cysts were prepared and decapsulated [29]. Briefly, 850 μl of NaOH (32%) and 12 ml of NaOCl (50%) were added to the suspension of hydrated cysts to facilitate decapsulation. The process was stopped after 3 min by adding 12 ml of Na_2_S_2_O_3_ (10 g/l). The decapsulated cysts were washed with autoclaved artificial seawater (ASW) (ISTA, Taiwan). For the experiments, the cysts were hatched for 24–28 hours at 25°C on a shaker at 80 rpm. After 24–28 hours of hatching, batches of 25 *Artemia* nauplii were counted and transferred to 6 cm petri dishes containing 10 ml of autoclaved ASW. Finally, the dishes were returned to the incubator and kept at 25°C [9, 30].

The 25 *Artemia* nauplii were collected and transferred to a 6 cm petri dish containing 10 ml ASW, and infected with different concentrations (2.5 × 10^9^, 5.0 × 10^9^, or 1.0 × 10^10^ CFU) of *V*. *parahaemolyticus* strain KX-V231 or YAS1206-16. The control group of *Artemia* nauplii was not infected with *V*. *parahaemolyticus*. After incubation at 25°C for 72 hours, the survival rates of *Artemia* nauplii were recorded [31]. The experiments were conducted in triplicate.

To assay the protection of O-741 bacterium against *V*. *parahaemolyticus* using the *Artemia* nauplii model, groups of 25 *Artemia* nauplii were incubated with different concentrations (1 × 10^8^, or 1 × 10^9^ CFU) of O-741 bacterium, then infected with 5 × 10^9^ CFU of *V*. *parahaemolyticus* strain KX-V231 or YAS1206-16 in 10 ml ASW at 25°C. After incubation for 72 hours, the survival rates of *Artemia* nauplii were recorded. The control group of *Artemia* nauplii was not incubated with O-741 bacterium and infected with *V*. *parahaemolyticus* strains. The experiments were conducted in triplicate.

### Evaluation of the stability and antimicrobial activity of O-741 CFS

The antimicrobial activities of O-741 CFS, which had been subjected to different stress treatments, were determined by well diffusion assays against *V*. *parahaemolyticus* strains KX-V231 or D/4.

To determine thermal stability, the CFS was heated at 37°C, 60°C, 80°C or 100°C for 30 or 60 min. The CFS was also digested by lysozyme (Sigma—Aldrich, St. Louis, MA, USA), proteinase K (Sigma—Aldrich, St. Louis, MA, USA), pronase (Sigma—Aldrich, St. Louis, MA, USA), catalase (Sigma—Aldrich, St. Louis, MA, USA), pepsin (Merck, USA) and trypsin-EDTA (Sigma—Aldrich, St. Louis, MA, USA) at 0.5 mg/ml concentration, at 37°C for 2 hours. The CFS was incubated at 37°C for 2 hours with acetone, acetonitrile, ethanol, ethyl acetate, ethyl ether, and methanol at concentrations of 10%. The CFS was adjusted to different pH values of 2, 4, 6, 8, 10, and 12 by HCl or NaOH, and incubated at room temperature for 1 hour. The CFS was also subjected to UV irradiation by being placed 75 cm from a 30W UV light source for 1 to 5 hours.

### Extraction and analysis of antimicrobial compounds

For extraction of antimicrobial compounds, the O-741 CFS was extracted with 1:1 (V:V) ethyl acetate by stirring for 2 hours at room temperature. The organic phase and aqueous phase were condensed with a refrigerated centrifugal concentrator, dried in a rotary evaporator, and dissolved with methanol or water, respectively. Then, the crude extracts were filtered through a 0.22 μm polyethersulfone membrane (Merck Millipore, Ireland).

To estimate the antimicrobial activities of crude extracts, disk diffusion assays were used. Briefly, 10 μl crude extracts from the organic layer or the aqueous layer were dropped onto a 6 mm paper disk on a LB-3% NaCl plate with a bacterial lawn. Then, the plates were incubated at 37°C for 16–18 hours. The antimicrobial activities were measured as diameter (mm) of the inhibition zones.

The fractions were then subjected to UPLC-MS/MS analysis. An Agilent 1290 Infinity II ultra-performance liquid chromatography (UPLC) system (Agilent Technologies, Palo Alto, CA, USA) coupled online to the Dual AJS electrospray ionization (ESI) source of an Agilent 6545XT quadrupole time-of-flight (Q-TOF) mass spectrometer (Agilent Technologies, Palo Alto, CA, USA) was used in this experiment. The samples were separated using an ACQUITY UPLC BEH C18 column (1.7 μm, 2.1 × 100 mm, Waters Corp., Milford, MA, USA). The mobile phases were ddH_2_O (eluent A) and acetonitrile (eluent B), both eluents had 0.1% formic acid. The column temperature was 40°C. The instrument was operated in positive full-scan mode.

The effective compounds were eluted according to the following linear gradient: first, starting at 80% eluent A and 20% eluent B, eluent A was linearly decreased to 0% with an increase of eluent B to 100% in 23 min and then maintained for 3 min at a flow rate of 0.3 ml/min.

### Bactericidal effect of antimicrobial compounds against *V*. *parahaemolyticus*

To determine the bactericidal effect on *V*. *parahaemolyticus*, different concentrations of the organic extracts from O-741 CFS (50, 100, or 200 μg/ml) were added to 5 ml Mueller Hinton broth (BD Difco, Detroit, MI, USA, DF0757-17-6) with 1 × 10^9^ CFU of *V*. *parahaemolyticus* strains KX-V231, D/4, or YAS1206-16, and incubated at 37°C for different times (0, 2, 4, 6, and 8 hours). The survival bacteria were enumerated using the dilution plate count method on a LB-3% NaCl plate and incubated at 37°C for 16 hours [32].

### Scanning electron microscopy of *V*. *parahaemolyticus* cells

The *V*. *parahaemolyticus* cells treated with organic extract of O-741 CFS at 0 μg/ml or 50 μg/ml were examined by Scanning Electron Microscopy (SEM). In addition, the bacterial cells were treated with methanol as control. Then, the cells were harvested by centrifugation and fixed in 4% paraformaldehyde and 2.5% glutaraldehyde, post-fixed in 1% osmium tetroxide, dehydrated through ethanol solutions, and dried in CO_2_ medium with a critical point dryer (Hitachi HCP-2). After coating with gold/palladium, observation was performed under a field emission scanning electron microscope (FE-SEM, Hitachi S-4700) [33].

### Statistical analysis

Triplicate experiments were performed in this study, and the data about the bacterial growth experiments were measured in triplicate. The data were analyzed by using t-test at a significance level of α = 0.05, using SPSS for Windows version 11.0 (SPSS, Chicago, IL, USA).

## Results

### Screening and identification of antimicrobial bacteria

**Fig 1:**
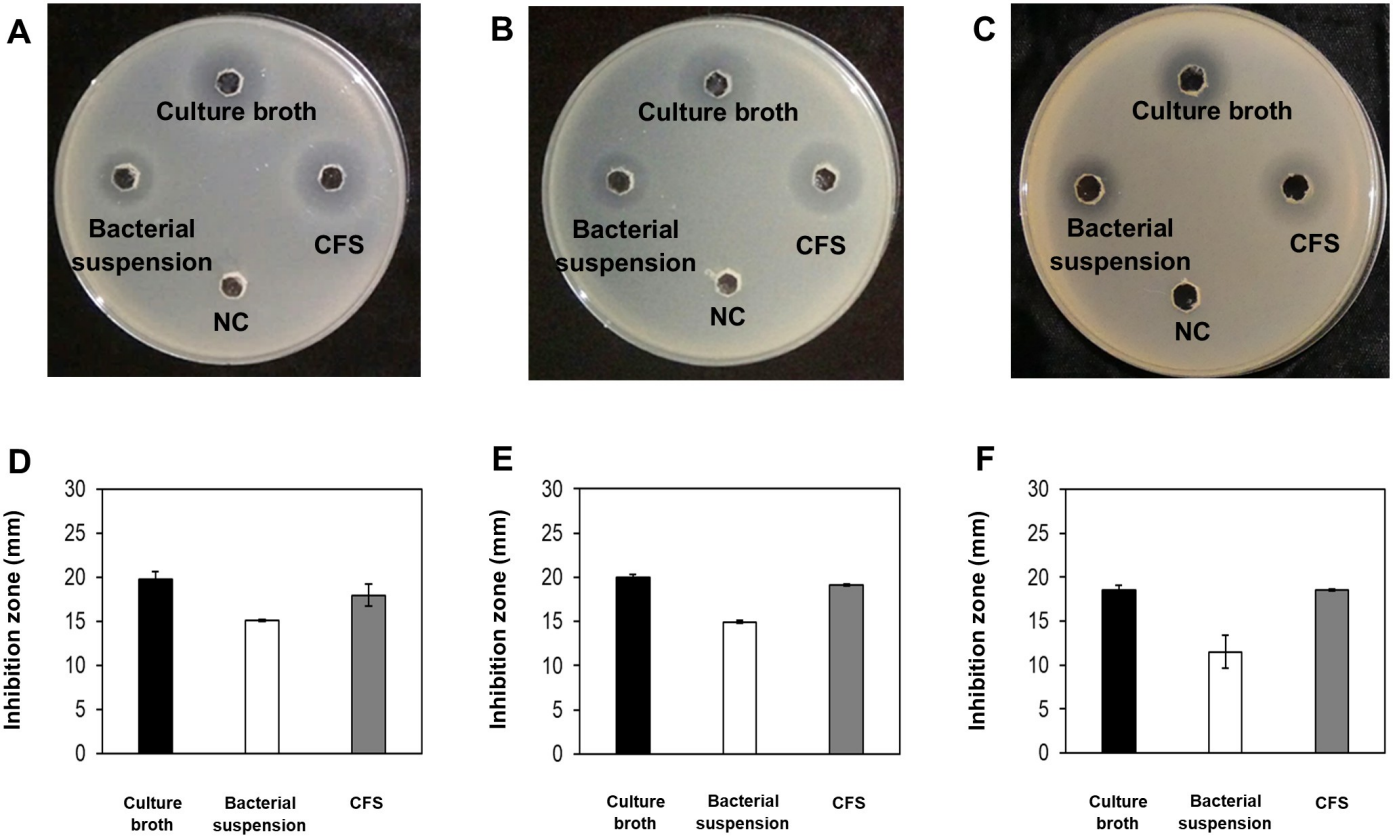
Antimicrobial activities of isolated O-741 bacterium revealed by well diffusion assays. The O-741 bacterial culture broth, bacteria suspension and cell-free supernatant (CFS) were loaded into the wells of LB-3% NaCl plates inoculated with different strains of *V*. *parahaemolyticus*. Then, the plates were incubated at 37°C for 24 hours. The results of well diffusion assays showed antimicrobial activities against *V*. *parahaemolyticus* strains KX-V231 (A), D/4 (B), or YAS1206-16 (C). The inhibition zones of O-741 bacterium against *V*. *parahaemolyticus* strains KX-V231 (D), D/4 (E), or YAS1206-16 (F) are shown. NC, LB broth was added as the negative control. Data shown are the mean in mm ± SE from three independent experiments.

For selection of bacteria against *Vibrio* species, a total of 1,545 bacterial isolates were screened and characterized by spot inoculation against *V*. *parahaemolyticus*, *V*. *harveyi*, and *V*. *vulnificus*. The results revealed that the isolate O-741 bacterium had obvious antimicrobial activities, which was further confirmed by well diffusion assays against *V*. *parahaemolyticus* strains KX-V231, D/4 and YAS1206-16 (Fig 1).

For identification of O-741 bacterium, genome DNA was extracted and the 16S rRNA, *gyrA*, and *rpoB* genes were amplified by PCR (S1 Fig) and sequenced. The analysis of BLAST matching showed that the nucleotides sequences of 16S rRNA, *gyrA*, and *rpoB* genes had a high similarity to the *Bacillus subtilis*. The phylogenetic trees were constructed, and the results showed that the strain O-741 clustered with *B*. *subtilis* strains (S2 Fig).

### Evaluation of the antimicrobial activities of O-741 bacterium

**Fig 2:**
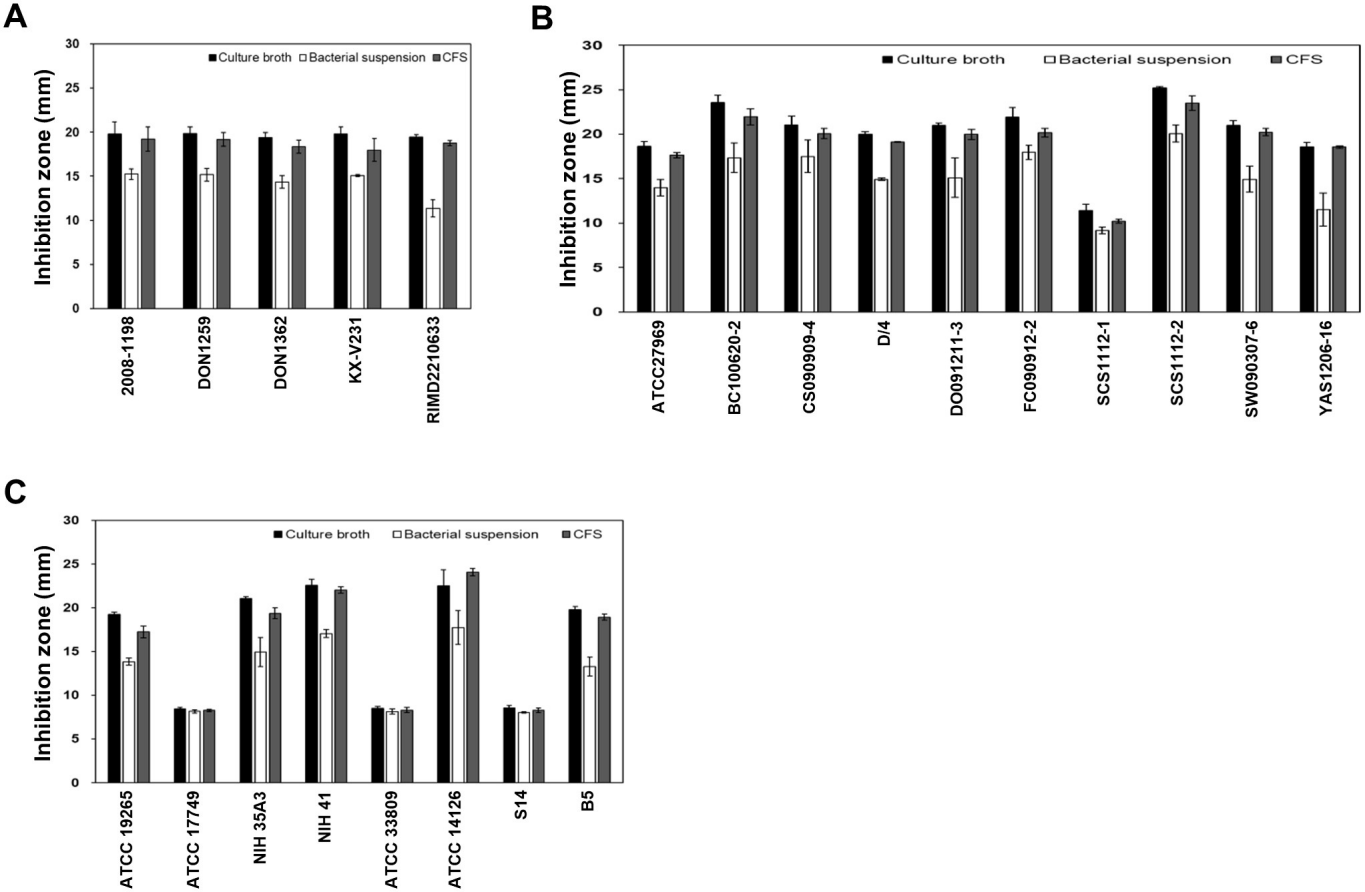
Antagonistic activities of O-741 bacterium were evaluated by well diffusion assays. (A) The antagonistic activities of O-741 bacterium on 5 clinical isolates of *V*. *parahaemolyticus*. (B) The antagonistic activities of O-741 bacterium on 10 environmental isolates of *V*. *parahaemolyticus*. (C) The antagonistic activities of O-741 bacterium on 8 isolates of *Vibrio* spp.. Data shown are the mean in mm ± SE from three independent experiments.

In order to determine the antimicrobial activities, the bacterial culture broths, bacterial suspensions, and cell-free supernatants (CFS) from O-741 bacterium (24-hour culture) were assayed against 5 clinical isolates and 10 environmental isolates of *V*. *parahaemolyticus*. The results showed that the O-741 bacterium had strong antimicrobial activity against *V*. *parahaemolyticus*. Further investigation of the antagonistic spectrum showed that O-741 bacterium had high antimicrobial activities against 8 isolates from different *Vibrio* species. These results indicate that O-741 bacterium exhibited broad-spectrum antimicrobial activity against *Vibrio* species (Fig 2).

### *In vivo* challenge using an *Artemia* nauplii model

We used the *Artemia* nauplii model to investigate the *in vivo* infection of *V*. *parahaemolyticus* strains KX-V231 and YAS1206-16 in an aquatic environment. Seventy-two hours post-infection by these *V*. *parahaemolyticus* strains, survival of *Artemia* nauplii was markedly reduced thus demonstrating the virulence of these two *V*. *parahaemolyticus* strains in this model (S3 Fig).

**Fig 3:**
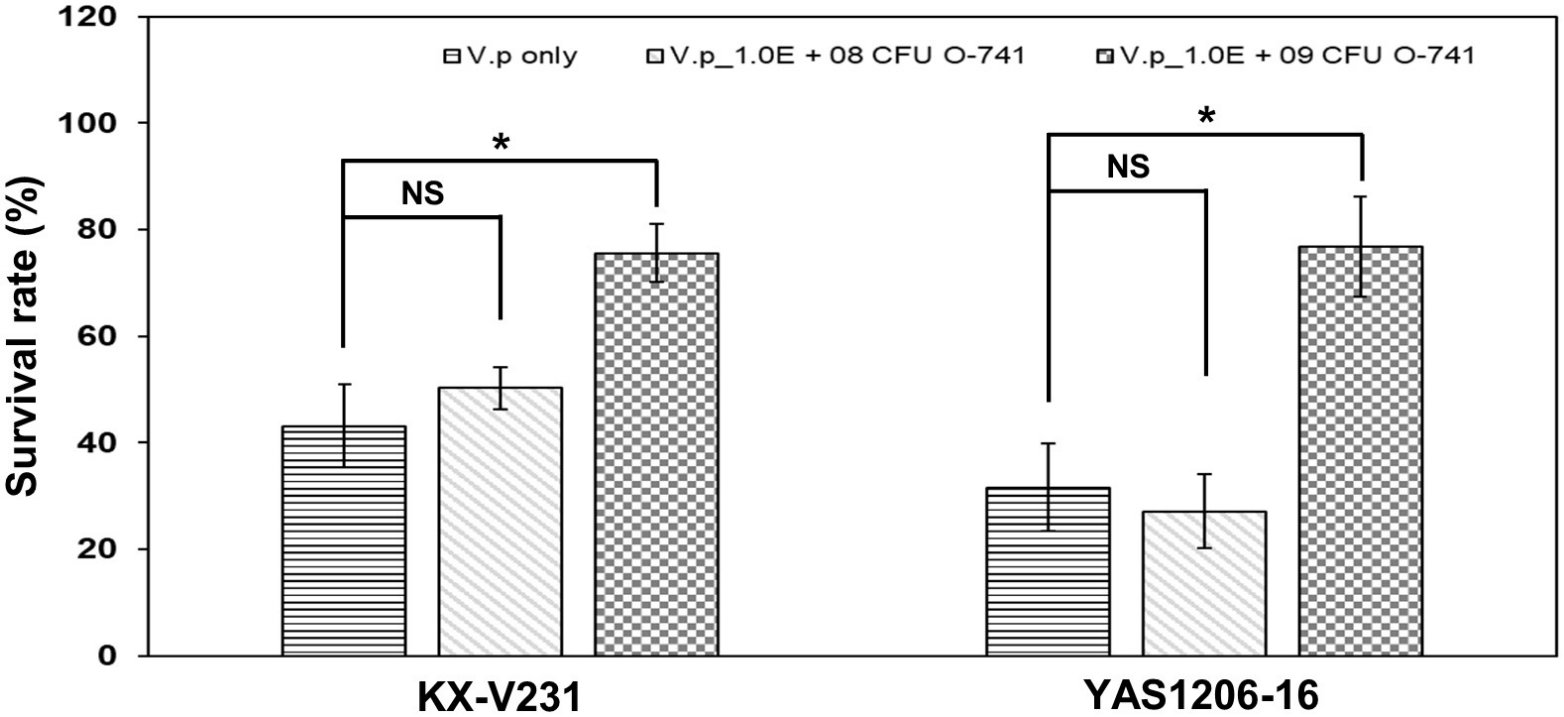
Protective activities of O-741 bacterium on the survival of *Artemia* nauplii infected with *V*. *parahaemolyticus*. Different concentrations (0, 1 × 10^8^, and 1 × 10^9^ CFU) of O-741 bacterium were incubated with *Artemia* nauplii (n = 25). Then, the nauplii were infected with 5 × 10^9^ CFU of *V*. *parahaemolyticus* strains KX-V231 or YAS1206-16. The survival rates were recorded after 72 hours. Data shown are the mean ± SE from three independent experiments. Unpaired t-tests were used to calculate P values. (NS: no statistical significance; *, p < 0.05).

In the *in vivo* challenge experiments, the *Artemia* nauplii were incubated with O-741 bacterium, and subsequently infected with *V*. *parahaemolyticus* strains KX-V231 or YAS1206-16. The survival rates of *Artemia* nauplii were significantly increased in groups pre-incubated with O-741 bacterium (Fig 3).

### Extraction and characterization of the O-741 CFS

**Fig 4:**
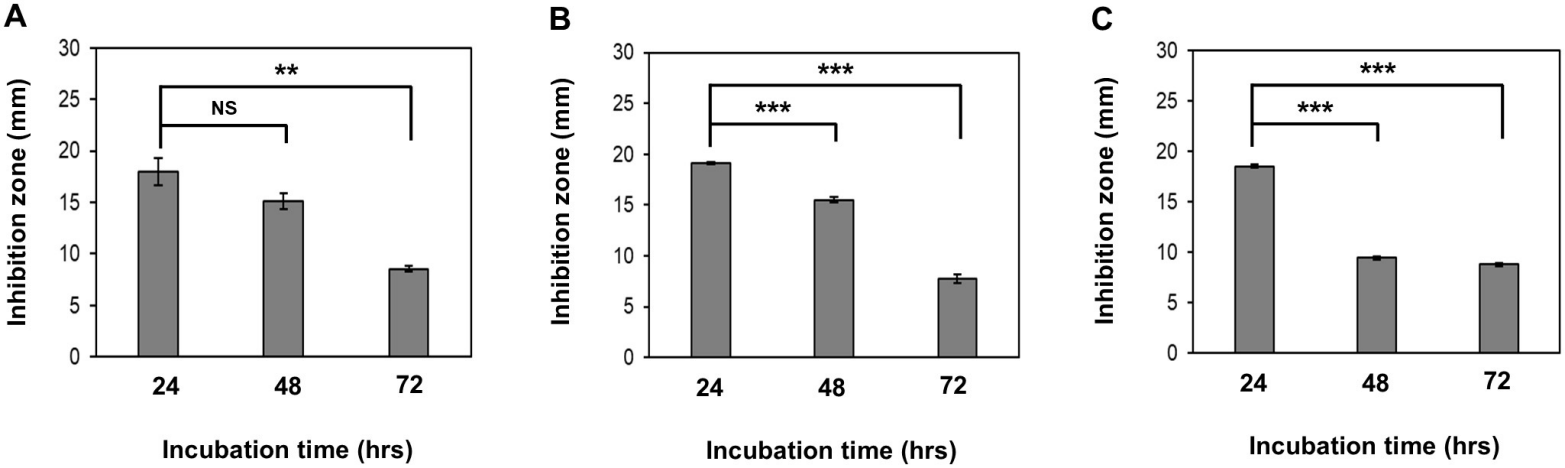
Inhibitory activities of O-741 CFS on *V*. *parahaemolyticus* at different incubation times. After culture for 24, 48, and 72 hours, the O-741 CFS was collected. The inhibitory activities of O-741 CFS on *V*. *parahaemolyticus* strains KX-V231 (A), D/4 (B), or YAS1206-16 (C) were evaluated. Data shown are the mean ± SE from three independent experiments. Unpaired t-tests were used to calculate P values. (NS: no statistical significance; **, p < 0.01; ***, p < 0.001).

After different culture times (24, 48, and 72 hours), the O-741 CFS were collected and the antimicrobial activities were examined by well diffusion assays. The O-741 CFS showed inhibitory activities against *V*. *parahaemolyticus* strains KX-V231, D/4, and YAS1206-16. The O-741 CFS from 24-hour culture exhibited the most significant inhibitory activities as compared to CFS from 48- or 72-hour culture (Fig 4).

The inhibitory activity of the O-741 CFS from 24-hour culture was characterized with indicator *V*. *parahaemolyticus* strains KX-V231 and D/4. The results showed high stability against different environmental stresses (S1 Table). The CFS was thermally stable. After being heated at 60°C for 60 min, 84.16 or 82.26% of the inhibitory activity remained when assayed with *V*. *parahaemolyticus* strains KX-V231 or D/4, respectively. When the temperature was increased to 100°C, more than 40% activity remained. The CFS was also resistant to digestion by different enzymes, since none of the tested enzymes (lysozyme, proteinase K, pronase, catalase, pepsin, and trypsin-EDTA) reduced the activities to lower than 90% relative to the untreated control. The activities of the CFS were stable to most of these organic solvents; acetone was the only solvent to decrease the activity to lower than 70%. In addition, the activities of the CFS were stable to a wide range of acidity treatments (pH 2 to pH 12), and UV irradiation.

For examination of the antimicrobial compound, the O-741 CFS from 24-hour culture was prepared, and extracted by ethyl acetate. After drying, the organic layer and aqueous layer were re-dissolved in methanol and ddH_2_O, respectively. Then, the crude extracts from the organic layer or the aqueous layer were assayed for antimicrobial activity. The results showed that the antimicrobial activity of crude extracts from the organic layer were markedly stronger than those from the aqueous layer, whereas 0.5 mg/ml crude extract from the aqueous layer showed 9.1–10.4 mm inhibition zones in these two indicators, and 0.05 mg/ml crude extract from organic layer showed 14.7–15.6 mm inhibition zones (Table 3).

**Table 3:** Antimicrobial activities of crude extracts from organic layer or aqueous layer.

		Size of inhibition zone (mean in mm ± SE)	
Crude extract	Concentration (mg/ml)	KX-V231	D/4
Organic layer	0.05	14.70 ± 0.53***	15.58 ± 0.55**
	0.025	11.00 ± 0.63***	12.76 ± 0.53**
	0.0125	7.77 ± 0.31**	9.26 ± 0.54*
	0.00625	6.80 ± 0.09**	7.02 ± 0.17*
	0.003125	6.50 ± 0.12^NS^	6.45 ± 0.07^NS^
	Control (methanol)	6.40 ± 0.06	6.50 ± 0.11
Aqueous layer	1	13.24 ± 0.30***	12.62 ± 0.61***
	0.5	10.41 ± 0.10***	9.05 ± 0.47**
	0.25	7.90 ± 0.13**	7.49 ± 0.19**
	0.125	6.55 ± 0.15*	6.30 ± 0.04^NS^
	0.0625	6.15 ± 0.02^NS^	6.24 ± 0.06^NS^
	Control (ddH_2_O)	6.18 ± 0.04	6.16 ± 0.12

Data shown are the mean in mm ± standard error (SE) from three independent experiments. Unpaired t-tests were used to calculate P values. (NS: no statistical significance; *, p < 0.05; **, p < 0.01; ***, p < 0.001, compared to the control).

### Analysis of antimicrobial compounds from the organic extracts

**Fig 5:**
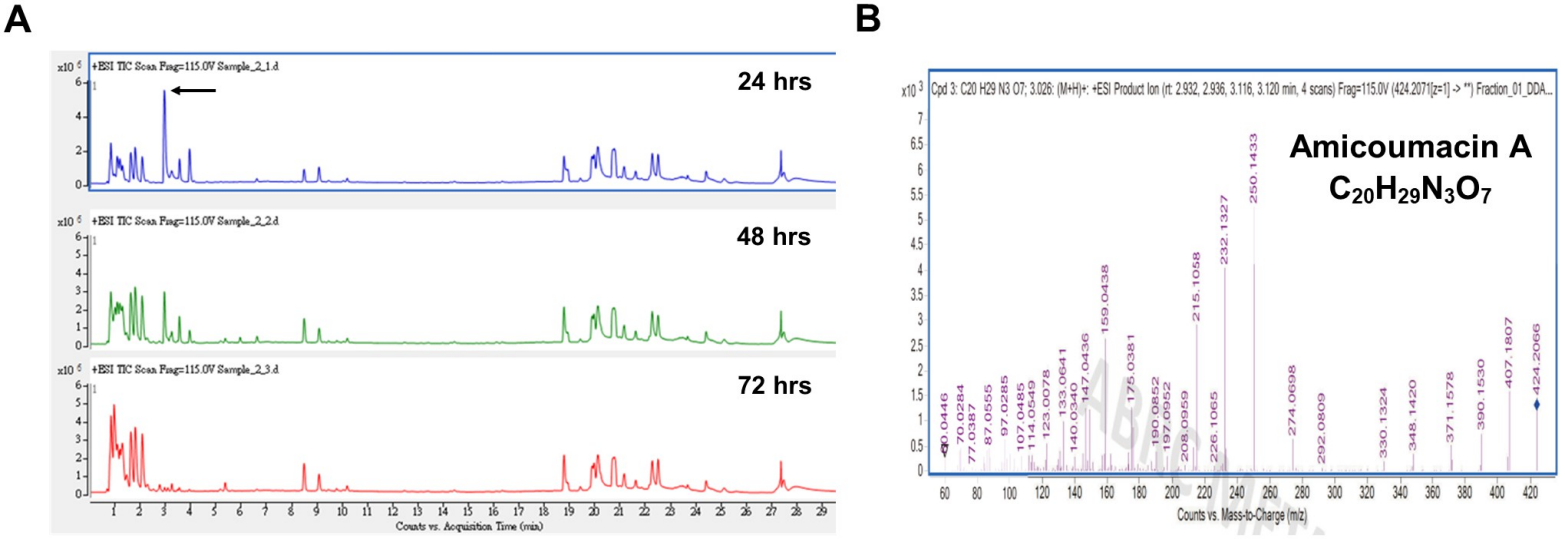
Extraction and characterization of antimicrobial substances from organic extracts. The crude extracts from organic layer were analyzed by UPLC-MS/MS. (A) The spectrum shows the mass of peptides and the dominant peak (arrow). With prolonged culture time, the dominant peak declined. (B) The fragmentation pattern at m/z 424 Da corresponds to the compound amicoumacin A.

The organic extracts from the 24, 48, and 72-hour O-741 cultures showed declined antimicrobial activity with prolonged culture time (Fig 4). In addition, analysis of organic extracts by UPLC-MS/MS spectrometry showed that the spectrum presented mass of peptides, and level of the dominant peak declined at 48, and 72 hours relative to 24 hours culture (Fig 5A). Furthermore, the fragmentation pattern at m/z 424 Da corresponded to amicoumacin A (Fig 5B; Table 4) [11, 33]. These results indicated that O-741 bacterium can produce amicoumacin A and exhibit antimicrobial activities.

**Table 4:** The UPLC-MS/MS data of compounds detected in organic extract of the O-741 CFS from 24-hour culture.

Retention time (min)	m/z value	Molecular formula	Compound name
1.072	105.0696	C_8_H_8_	Styrene
1.584	183.0919	C_12_H_10_N_2_	Harman
3.026	424.2078	C_20_H_29_N_3_O_7_	Amicoumacin A
10.19	281.1385	C_15_H_20_O_5_	Betaxolol(deaminated)
10.198	135.0808	C_9_H_10_O	Benzyl methyl ketone
10.387	119.0857	C_9_H_10_	alpha-Methylstyrene
14.355	279.1587	C_16_H_22_O_4_	Dibutyl phthalate
20.071	338.3428	C_22_H_43_NO	N-cyclohexanecarbonylpentadecylamine
22.126	391.2846	C_24_H_38_O_4_	7b-Hydroxy-3-oxo-5b-cholanoic acid
22.484	391.2852	C_24_H_38_O_4_	7b-Hydroxy-3-oxo-5b-cholanoic acid
22.519	338.3429	C_22_H_43_NO	N-cyclohexanecarbonylpentadecylamine
22.851	391.2846	C_24_H_38_O_4_	Dioctyl phthalate
22.877	338.342	C_22_H_43_NO	N-cyclohexanecarbonylpentadecylamine
23.218	391.284	C_24_H_38_O_4_	Dioctyl phthalate
23.459	251.0813	C_15_H_10_N_2_O_2_	6-Anilino-5,8-quinolinedione
23.538	127.0757	C_7_H_10_O_2_	1-Cyclohexene-1-carboxylic acid
23.559	155.0703	C_8_H_10_O_3_	2,6-Dimethoxyphenol
23.564	267.1958	C_16_H_26_O_3_	Juvabione
23.572	285.2062	C_16_H_28_O_4_	(10S)-Juvenile hormone III diol
23.813	391.284	C_24_H_38_O_4_	7b-Hydroxy-3-oxo-5b-cholanoic acid
24.18	721.48	C_41_H_69_O_8_P	PA(18:1(9Z)/20:5(5Z,8Z,11Z,14Z,17Z))
24.433	447.3458	C_28_H_46_O_4_	Didecyl phthalate
27.239	122.0967	C_8_H_11_N	2,5-Xylidine

**Fig 6:**
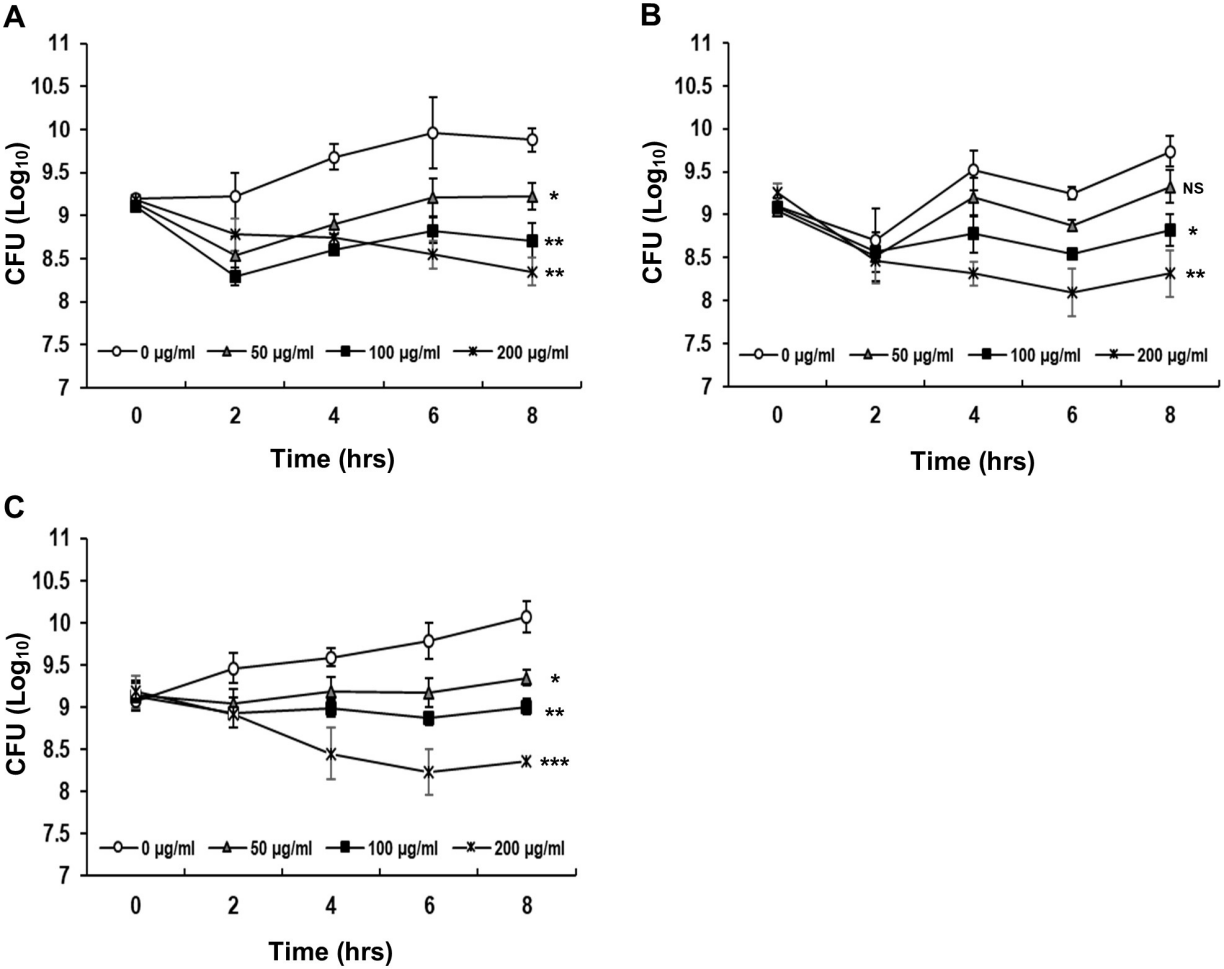
Bactericidal effects of antimicrobial substances on *V*. *parahaemolyticus*. The 50, 100 or 200 μg/ml of organic extracts from 24-hour culture were incubated with bacterial cultures of *V*. *parahaemolyticus* strains KX-V231 (A), D/4 (B), or YAS1206-16 (C) for 8 hours. After treatment, the growth of colonies was counted and recorded. Unpaired t-tests were used to calculate P values (NS: no statistical significance; *, p < 0.05; **, p < 0.01; ***, p < 0.001).

The bacterial cultures of *V*. *parahaemolyticus* strains KX-V231, D/4, or YAS1206-16 were incubated with 50, 100, or 200 μg/ml of organic extracts from the 24-hour culture for 8 hours. Survival levels of these *V*. *parahaemolyticus* strains significantly decreased along with the increase in organic extract amount (Fig 6). These results demonstrated that the organic extract from 24-hour culture contained the antimicrobial amicoumacin A and had bactericidal activities.

### Effect of antimicrobial compounds on cell morphology of *V*. *parahaemolyticus*

**Fig 7:**
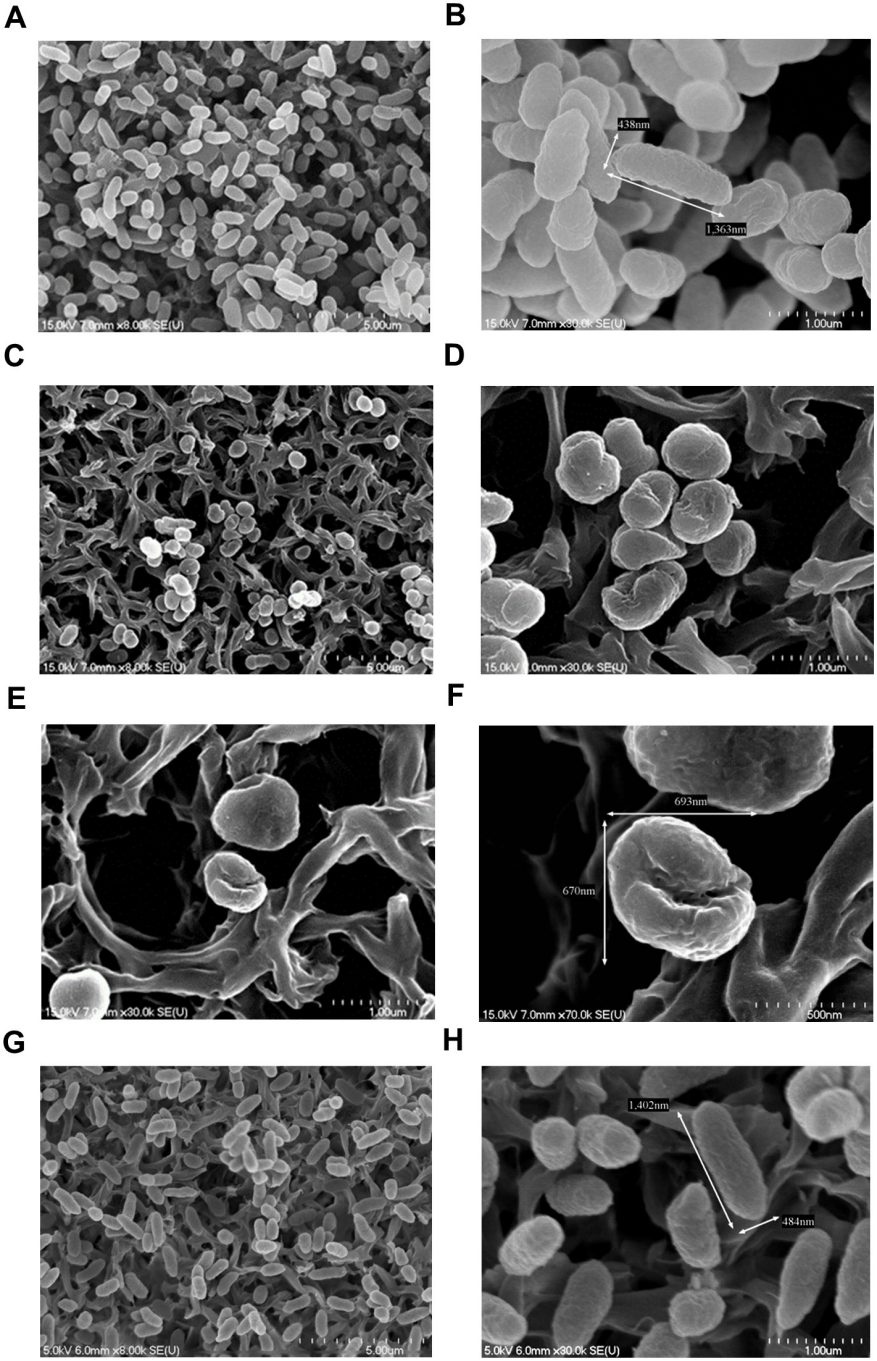
SEM micrographs of *V*. *parahaemolyticus* cells. The *V*. *parahaemolyticus* KX-V231 cells were treated with 0 μg/ml (A, B) or 50 μg/ml (C, D, E, F) of organic extract containing antimicrobial substances for 8 hours. In addition, the cells were treated with methanol as control (G, H). Damage to the surface structure is observed obviously in the cells treated with organic extract.

Following treatment with organic extract containing antimicrobial amicoumacin A, the morphology of *V*. *parahaemolyticus* cells was observed by SEM (Fig 7). The cells treated with organic extract at 0 μg/ml were rod-shaped (1,363 × 438 nm) with a smooth cell surface (Fig 7A and 7B). However, the cells treated with organic extract at 50 μg/ml were mostly coccoid-shaped (693 × 670 nm) with irregular collapse that formed cavities in the cell surface (Fig 7C–7F). As a control, the cells were treated with methanol and maintained the rod-shaped morphology (Fig 7G and 7H). These observations indicate that the organic extract can disrupt the cell membrane and the cell wall of *V*. *parahaemolyticus* cells.

## Discussion

Vibriosis is an illness caused by infection with *Vibrio* bacteria. Overuse of chemicals and antibiotics in controlling vibriosis can result in environmental pollution, and drug resistance issues [34]. Drug-resistant microorganisms can greatly enhance the risk of infection, and have enormous impacts on aquaculture and human health [35]. The use of biocontrol agents (BCA) is considered to be an environmentally-friendly approach to lower the threat of pathogenic *Vibrio* bacteria.

The *Bacillus* strains are appropriate biocontrol agent candidates for prevention of bacterial infections. Many *Bacillus* species have been proven to be safe, and some strains are used as probiotics for human and animal consumption [16]. Recently, *Bacillus* species have been used to inhibit aquatic pathogenic bacteria, including *V*. *parahaemolyticus* [11, 17], *V*. *anguillarum* [33], *V*. *alginolyticus* [36], *V*. *cholerae* [37], *V*. *harveyi* [38], and *V*. *vulnificus* [33]. In this study, we screened and identified a *Bacillus subtilis* strain O-741 from an aquaculture environment. The O-741 bacterium and its CFS exhibited strong antimicrobial activities against 23 strains of 7 *Vibrio* species (Table 1). In addition, the O-741 bacterium was able to protect *Artemia* nauplii from infection with *V*. *parahaemolyticus*. The characteristics of the O-741 bacterium show a wide antagonistic spectrum and thus, this bacterium may be a good candidate for use in prevention of *Vibrio* bacterial infection.

Several pieces of research have reported the activity of the antimicrobial compounds in the CFS produced by *B*. *amyloliquefaciens* [39], *B*. *pumilis* [33, 40] and *B*. *subtilis* strains [11, 28] under physical and chemical treatments. The results of stability assays strengthen the evidence for the possible useful application of CFSs as fish feed additives [41]. In this study, the stability and antimicrobial activity of the O-741 CFS were also investigated. The O-741 CFS was found to have inhibitory activity and be highly stable to heat, enzymes, organic solvents, pH, and UV treatments (S1 Table). The results of the stability assays of O-741 CFS further support its possible efficient application under different environmental conditions.

In this study, the active antimicrobial compound in the O-741 CFS was identified to be amicoumacin A. Amicoumacin A was isolated for the first time from *Bacillus pumilus* by Itoh et al. [42]. This compound has also been found in other *Bacillus* strains [11, 33, 43–45]. Amicoumacin A exhibits inhibitory activities against different bacterial pathogens, such as *Helicobacter pylori*, methicillin-resistant *Staphylococcus aureus* (MRSA) and *Vibrio* species [33, 46, 47]. The anti-inflammatory and antitumor effects of amicoumacin A have also been described [42]. We also showed that the amicoumacin A in O-741 CFS highly inhibited *V*. *parahaemolyticus* and some other pathogenic *Vibrio* bacteria that are responsible for *Vibrio* diseases in finfish, shellfish and shrimp [15, 48]. The broad antimicrobial spectrum of amicoumacin A may make it an effective prophylactic/therapeutic agent for *Vibrio* diseases in aquaculture.

Previous studies have indicated that amicoumacin A is the major metabolite accumulated at early incubation time points, but declines within 24 hours, and then appears as other amicoumacin derivatives [49, 50]. However, the derivatives of amicoumacin have weak antimicrobial activities [50]. A similar accumulation, decline, and derivation of amicoumacin A was observed in O-741 CFS with changes in antimicrobial activity (Figs 4 and 5). The action of amicoumacin A has been reported in *V*. *vulnificus*, in which the cell was damaged by membrane poration [33]. Furthermore, Lama et al. found that the amicoumacin A regulates the autolysis and activity of murein hydrolase in methicillin-resistant *Staphylococcus aureus* (MRSA) [47]. The murein hydrolase is involved in turnover of peptidoglycan in the cell wall and daughter cell separation after cell division. Amicoumacin A exhibits antimicrobial activity through reduction of murein hydrolase activity. A recent study suggested that amicoumacin A can interfere with translation by locking the mRNA in the ribosome and be a protein synthesis inhibitor [51, 52]. These reports indicate that amicoumacin A uses multiple mechanisms to inhibit the bacteria. Our findings demonstrate that exposure to the organic extract containing amicoumacin A resulted in the transformation of *V*. *parahaemolyticus* cells from rod-shaped to coccoid-shaped form (Fig 7). The observations suggest that amicoumacin A has the capability to impact cell membranes, cell walls, and potentially affect the cellular processes responsible for cell morphology. It is worth investigating the detailed molecular mechanism(s) behind this structural alteration caused by amicoumacin A.

In aquaculture, *V*. *parahaemolyticus* is one of the major pathogenic *Vibrio* bacteria leading to high rates of mortality of aquatic organisms and massive economic losses [53]. *Artemia* nauplii are aquatic invertebrates and the primary feed for farmed fish and shrimps [54]. In addition, they have been used as a model for examination of *V*. *parahaemolyticus* infection [9, 30]. Our study demonstrated that *Artemia* nauplii is a valid model to assay the infection of *V*. *parahaemolyticus* strains, and in this model the O-741 bacterium provided significant protection against this pathogen (Fig 3). These results indicate that the O-741 bacterium may have potential applications in aquaculture.

In conclusion, a novel *B*. *subtilis* O-741 bacterium was isolated and its antimicrobial activities against various pathogenic *Vibrio* bacteria including *V*. *parahaemolyticus*, *V*. *anguillarum*, *V*. *alginolyticus*, *V*. *chloreae*, *V*. *fluvialis*, *V*. *harveyi* and *V*. *vulnificus* were evaluated. The functional compound of O-741 and its action on *V*. *parahaemolyticus* were identified, and its suitability for application in aquaculture was also verified by the *Artemia* nauplii model. Furthermore, our findings suggest that the antagonistic O-741 bacterium may be candidate for preventing *Vibrio* bacterial infection in humans, and may aid in reducing the potential risk of disease transmission.

## Supporting information

S1 Fig PCR amplification of 16S rRNA, *gyrA*, and *rpoB* genes was performed for identification of the O-741 bacterium. Lane M, 100 bp DNA ladder (Thermo Scientific, Waltman, MA, USA); Lane 1, 16S rRNA PCR product; Lane 2, *gyrA* PCR product; Lane 3, *rpoB* PCR product.
(TIF)

S2 Fig Phylogenetic tree of *Bacillus subtilis* strain O-741 and its closest relatives based on 16S rRNA (A), *gyrA* (B), and *rpoB* (C) sequences. The phylogenetic trees were constructed by the neighbor-joining (NJ) method using MEGA6.0 software. The bootstrap values are shown at the branch points. Genbank accession numbers of the sequences are indicated in parentheses.
(TIF)

S3 Fig The survival rates of *Artemia* nauplii after infection for 72 hours with *V*. *parahaemolyticus*. Groups of 25 *Artemia* nauplii were infected with different concentrations of *V*. *parahaemolyticus* strains KX-V231 or YAS1206-16. The survival rates were recorded after 72 hours. Data shown are the mean ± SE from three independent experiments. Unpaired t-tests were used to calculate P values. (*, p < 0.05, **, p < 0.01, ***, p < 0.001, compared to blank).
(TIF)

S1 Table Effects of heat, enzymes, organic solvents, pH, and UV irradiation on inhibitory activities of the O-741 CFS from 24-hour culture. (DOCX)
