# Supplementary figures and images for: Isolation and characterization of an antimicrobial Bacillus subtilis strain O-741 against Vibrio parahaemolyticus
Source: PLoS One. 2024 Apr 4;19(4):e0299015. doi: 10.1371/journal.pone.0299015 (PMC10994408; doi:10.1371/journal.pone.0299015)

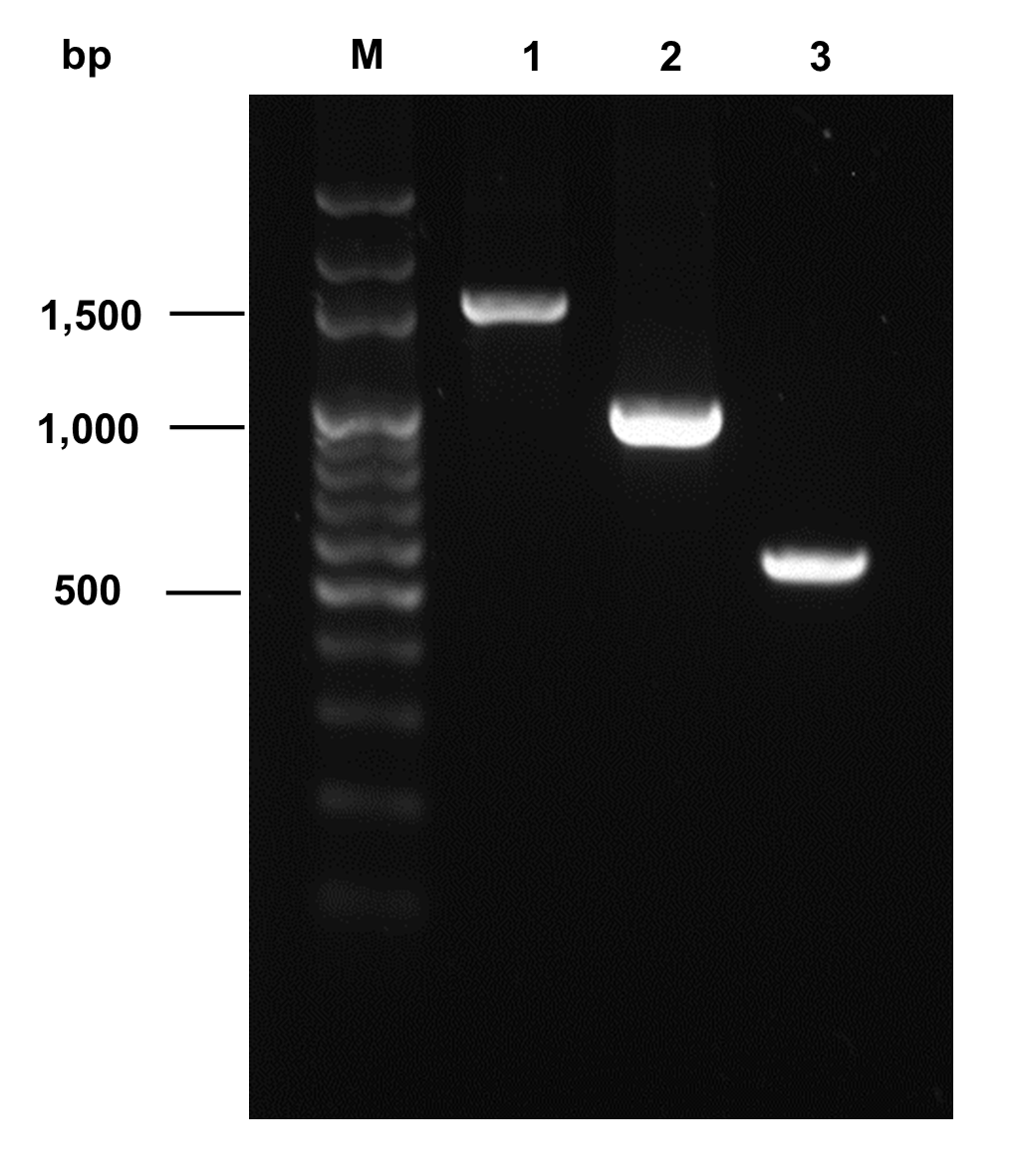

Supplement: S1 Fig — Lane M, 100 bp DNA ladder (Thermo Scientific, Waltman, MA, USA); Lane 1, 16S rRNA PCR product; Lane 2, gyrA PCR product; Lane 3, rpoB PCR product. (TIF) [file pone.0299015.s001.tif]

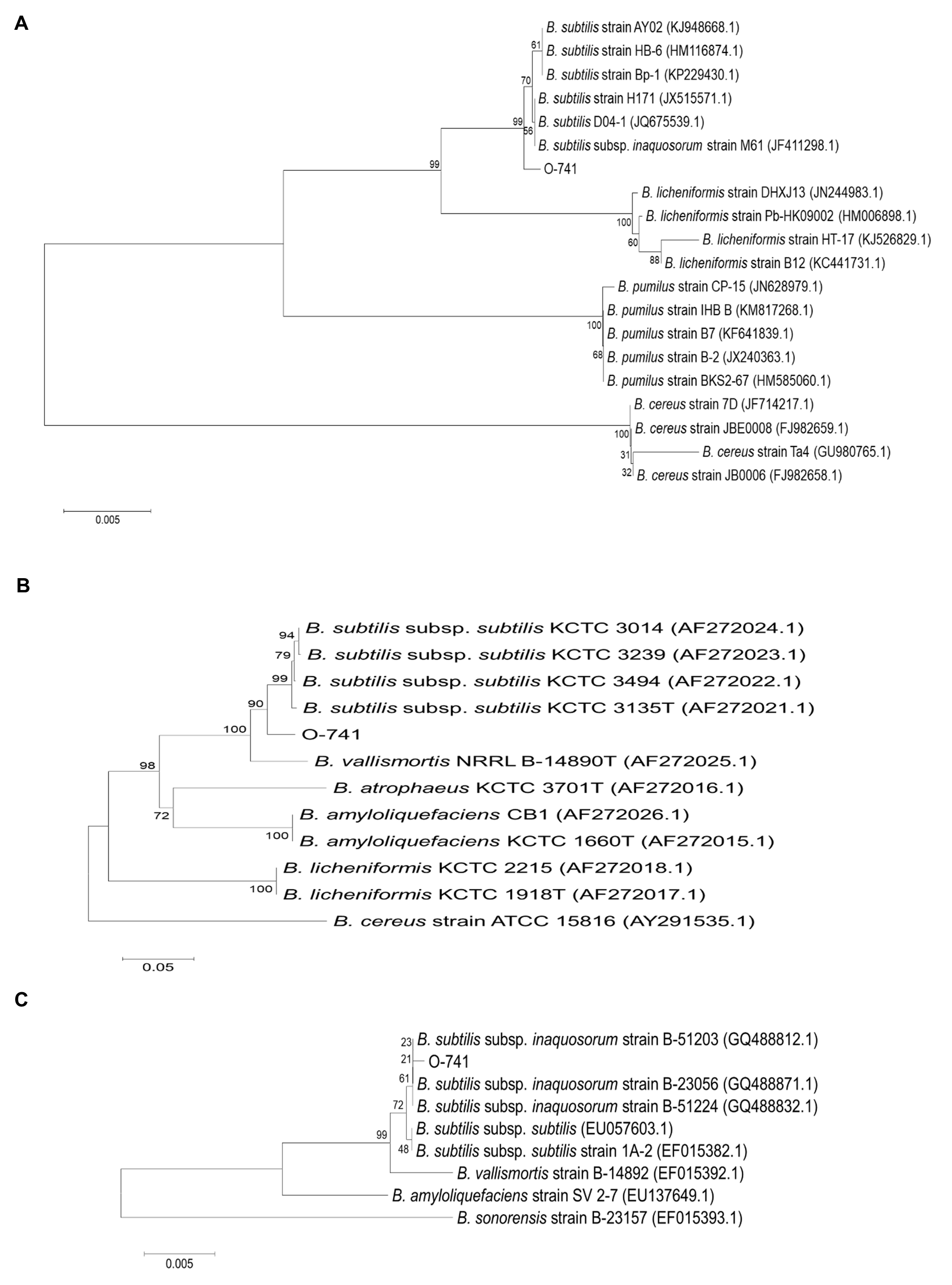

Supplement: S2 Fig — The phylogenetic trees were constructed by the neighbor-joining (NJ) method using MEGA6.0 software. The bootstrap values are shown at the branch points. Genbank accession numbers of the sequences are indicated in parentheses. (TIF) [file pone.0299015.s002.tif]

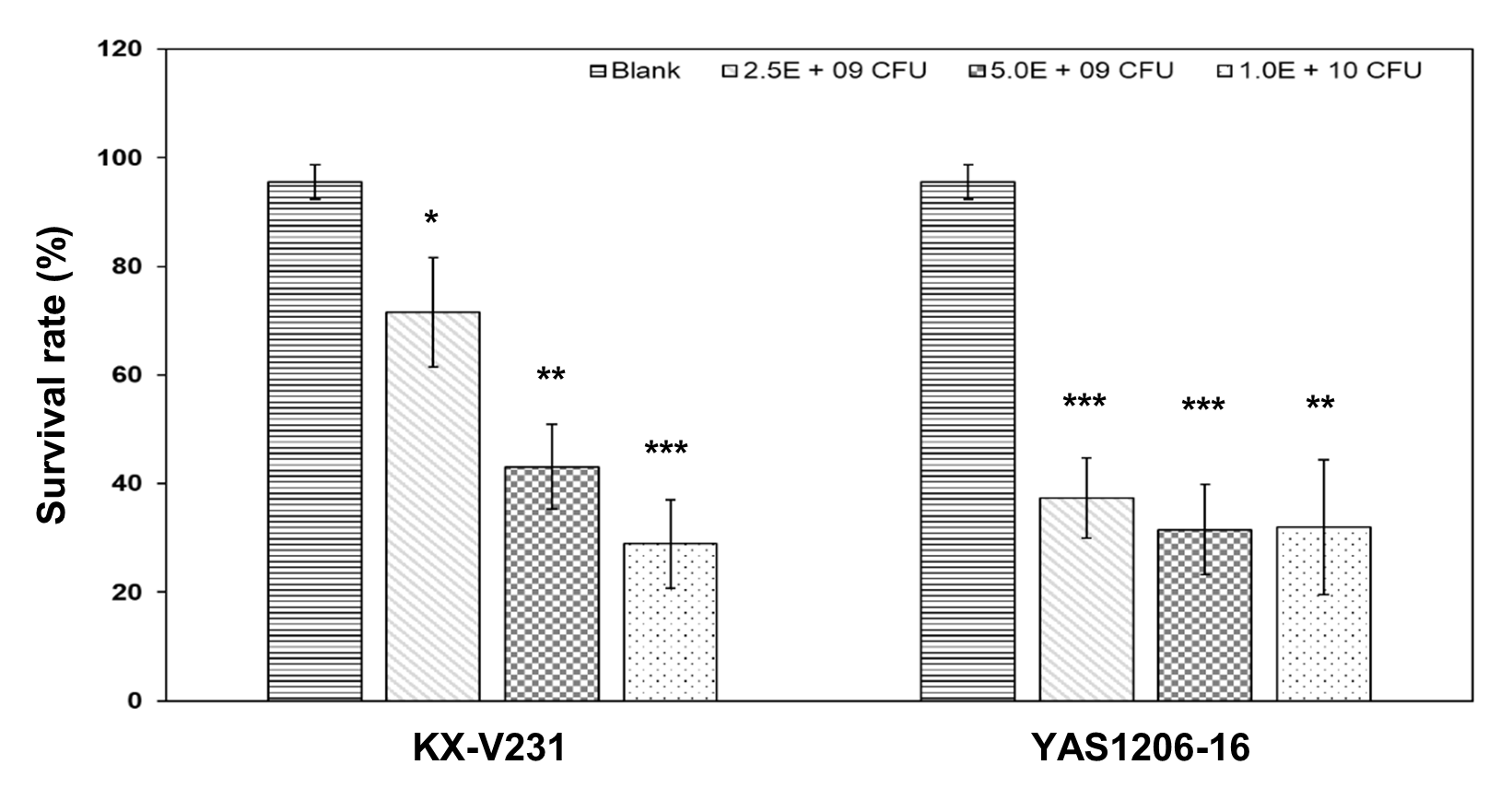

Supplement: S3 Fig — Groups of 25 Artemia nauplii were infected with different concentrations of V. parahaemolyticus strains KX-V231 or YAS1206-16. The survival rates were recorded after 72 hours. Data shown are the mean ± SE from three independent experiments. Unpaired t-tests were used to calculate P values. (*, p < 0.05, **, p < 0.01, ***, p < 0.001, compared to blank). (TIF) [file pone.0299015.s003.tif]
