# Supplementary material for: Isolation and characterization of an antimicrobial Bacillus subtilis strain O-741 against Vibrio parahaemolyticus
Source: PLoS One. 2024 Apr 4;19(4):e0299015. doi: 10.1371/journal.pone.0299015 (PMC10994408; doi:10.1371/journal.pone.0299015)
Supplement: S1 Table — (DOCX) [file pone.0299015.s004.docx]

**S1 Table. Effects of heat, enzymes, organic solvents, pH, and UV irradiation on inhibitory activities of the O-741 CFS from 24-hour culture.**

| Experiment | Treatment | Relative inhibitory activities (%)* | |
| --- | --- | --- | --- |
|  |  | KX-V231 | D/4 |
| Thermal stability | Control | 100.00 | 100.00 |
|  | 60°C, 30 min | 93.73 | 93.24 |
|  | 80°C, 30 min | 47.63 | 47.60 |
|  | 100°C, 30 min | 42.86 | 44.61 |
|  | 60°C, 60 min | 84.16 | 82.26 |
|  | 80°C, 60 min | 43.40 | 44.29 |
| Sensitivity to enzymes | Control | 100.00 | 100.00 |
| (0.5 mg/ml in 37°C, 2 h) | Lysozyme | 92.90 | 90.55 |
|  | Proteinase K | 92.20 | 88.39 |
|  | Pronase | 95.83 | 93.64 |
|  | Catalase | 94.23 | 88.12 |
|  | Pepsin | 95.12 | 94.13 |
|  | Trypsin-EDTA | 94.48 | 93.85 |
| Sensitivity to organic solvents | Control | 100.00 | 100.00 |
| 10% (v/v), 10 min | Acetone | 70.62 | 66.60 |
|  | Acetonitrile | 102.84 | 98.85 |
|  | Ethanol | 100.93 | 96.23 |
|  | Ethyl acetate | 97.34 | 95.57 |
|  | Ethyl ether | 99.99 | 101.84 |
|  | Methanol | 98.60 | 98.97 |
| pH stability | Control | 100.00 | 100.00 |
|  | pH 2 | 94.73 | 96.47 |
|  | pH 4 | 96.34 | 102.09 |
|  | pH 6 | 99.32 | 103.77 |
|  | pH 8 | 97.98 | 96.87 |
|  | pH 10 | 98.26 | 99.84 |
|  | pH 12 | 96.47 | 99.65 |
| UV irradiation | Control | 100.00 | 100.00 |
|  | UV, 1 h | 97.78 | 101.07 |
|  | UV, 3 h | 98.60 | 102.60 |
|  | UV, 5 h | 98.65 | 100.77 |

*The untreated CFS was used as a control. Relative inhibitory activities are compared to the control.
